# Supplementary material for: Chromothripsis during telomere crisis is independent of NHEJ, and consistent with a replicative origin
Source: Genome Res. 2019 May;29(5):737–49. doi: 10.1101/gr.240705.118 (PMC6499312; doi:10.1101/gr.240705.118)
Supplement: Supplemental Material [file supp_gr.240705.118_Supplemental_file_1.zip › contigs/annotated_contigs/DB53/contig.4.DB53_length_1360_mean_cov_11.3433823529.docx]

**DB53_length_1360_mean_cov_11.3433823529**

```
ATATTGCTCAGGCTGGTCTCAAACTCCTGGCCTCAAACA|ATCCTCC|TGCCTCAGACCCCCAAGTGCTGGACTTACAGGTTGAGCCAT
 >chr5:78949079-78949125 - E=1e-12 p=0e+00 >chr8:81074387-81075074 - E=0e+00 p=2e-02
TGTGCCAGGCTGTTTTGCCTGTTTTTGATCAGATTAGTTGTTGAGTTGTACGACTTTTTTATATATATGTTCTGGATATTAACCCTTTA

TTGGATGTTTGGTTTGCAAATATTTTCTCTTATTACGTAAATTGCCTTTTTATTGTCTTTTTTTTCTTTTCTCTTTTTAAGAGATTGAG

TCTTTCTCTTTTGTCCAGACTAGAGTGCAGTGATGCAATTCTAGCTCATTGTAGCCTTGAACTCCTGGGCTCAAGGAGTCCTTCTGCCT

CAGTCTCTCTAGTCATTTCCTTAAAAAAAAATTACAGAGCTTATAGGCTTCAATTTGTCTGTTTCCTAGAGAGGTTATAGATGTTATTA

TCCATTTGTATTTCCCTCTAGAGATTGTTACTTATCTACTTTGGGATCTTAGTGTGTCTTGCCTTCTCATTGTTCTCTGTCACCTTCCC

TCCTTGGCAAGTATTTACTTTTTCTATTTTGCTTATCATTTTGGTTATGTTGGATCTTTCAATGTTATTTTTATTAGCAAAATACAGTA

TTAGGAGCAAATATTTTCCAGGTGACATTTGGGGGTAATTTGCATGGCTTTCTGTGGCTTTCAGTACTTCCTTCTTGGTGTCTTTTCTG

TTTAGACAC|TTGTA|TTTTTAGTAGAGATGGGGTTTCACCATGTTGGTCAGGCTGGTCTCGAATTCCTGACCTCTTGATCCACCCGCC
          >chr8:75096455-75096717 + E=3e-138
TCGGCCTCCCAAAGTGTTGGGATTATAGGCAGGAGCCACCACGACTAGCCTTATTTATGTATCTTAATCAACAAAATGACTGGTACTTA

ACAAAGGAATCAGAAAATCTAAAAAAAAAACTATAACTTATGTATCTTAATCAAACCAGGAAGCAGCCACAGCAGCCTGAGTTCTGAAC

T|TC|CTCGGCCTCGGTGCCCACTCTGGCTGCACTTGAGGAGCCCTTCAGCCCGCCGCTGCACTGTGGGAGCCCCTCTCTGGGCTGGCT
  >chr8:75091877-75092261 - E=9e-213
GAGGCCGGAGCCGACTCCCTCTGCTTGCAGGGAGGTGTGGAGAGAGAGGCGCGGGCAGGAACCAAGGCTGCGCACAGCGCTCACGGGCC

ACTGCAAGTTCTGGGTGGGCGTGGGCTCCGCGGGCCCCGCACTCGGAGCCGCTGGCCCCGGGCAGTGAGGGGCTTATCACCCGGGCCAG

CAGCTGTGGAGGGTATGCCAGGTCCCCCAGCAGTGCCGGCTTGCCAGCACTGCACTCGAATTCTCACCGGGCCTCAGCTGCCTCCCCGC

AGGGCAGGGCTTGGGACCTGTAGCCCGCCAT
```
